# Trajectories and social determinants of child cognitive development: a prospective cohort study from infancy through middle childhood in Dhaka, Bangladesh

**DOI:** 10.1016/j.lansea.2024.100511

**Published:** 2024-11-23

**Authors:** Viviane Valdes, Eileen F. Sullivan, Fahmida Tofail, Lisa M. Thompson, Shahria H. Kakon, Talat Shama, Rashidul Haque, Charles A. Nelson

**Affiliations:** a Boston Children's Hospital (Division of Developmental Medicine), Harvard Medical School (Department of Pediatrics), Boston, MA, USA; b International Center for Diarrhoeal Disease Research (Nutrition Research Division), Dhaka, Bangladesh; c Emory University Nell Hodgson Woodruff School of Nursing, Emory University Rollins School of Public Health (Department of Environmental Health), Atlanta, GA, USA; d International Center for Diarrhoeal Disease Research (Infectious Diseases Division), Dhaka, Bangladesh; e Boston Children's Hospital (Division of Developmental Medicine), Harvard Medical School (Department of Pediatrics), Harvard Graduate School of Education, Cambridge, MA, USA

**Keywords:** Trajectories, Cognition, Maternal education, Food security, Poverty, Social determinants

## Abstract

**Background:**

Over a third of children globally do not meet their developmental potential, and children living in low and middle-income countries (LMICs) are most vulnerable. Understanding the contextual factors that influence cognitive development for children in LMICs is crucial to inform and develop interventions. We sought to characterize developmental trajectories of cognition in Bangladeshi children and identify salient social determinants.

**Methods:**

We used a longitudinal design and included 542 children living in Dhaka, Bangladesh. Social determinants (maternal and paternal education, housing risks, household assets, and food security) were assessed at baseline visits using a combination of oral interviews and home assessments. Cognitive development was assessed at 6 months, 2, 3, 4, 5, and 7 years. A total of 1397 cognitive assessments were completed across all participants. Growth curve models and mixed effect models were used.

**Findings:**

In children living above the poverty line, there was increasing deviation from expected cognitive scores from 6 months to 2 years (−12.85, *p* < 0.001) with stable scores from 2 to 7 years. For those below the poverty line, there were widening disparities from expectations in scores from 6 months to 4 years (−24.58, *p* < 0.001) with stable scores from 4 to 7 years. Higher levels of maternal education (*t* = 2.22, *p* = 0.03) and more food security (*t* = 4.48, *p* < 0.001) were protective for cognition longitudinally. Interaction effects between poverty level and maternal education and food security respectively were observed.

**Interpretation:**

Cognitive development trajectories showed increasing disparities from expectations in the first two years of life, with more pronounced and lasting effects through 4 years for children below the poverty line. Maternal education and food security had promotive/protective effects on longitudinal cognitive development scores for the full sample. Maternal education and food security had stronger effects on cognitive development for children living below the poverty line compared to those living above the poverty line.

**Funding:**

Funding for the study was provided by a grant from the 10.13039/100000865Bill and Melinda Gates Foundation (OPP1111625) to CAN.

**Research in context**

**Evidence before this study**

Poverty has long been associated with lower cognitive scores in both high-income countries (HICs) and LMICs. Being born into poverty, even in a HIC, has been linked to lower test scores at 3 years of age, and persistent exposure to poverty is linked to cumulative negative effects on cognitive development. There is emerging consensus that financial poverty is not the sole driver of poor cognitive outcomes. Instead, the link between poverty and cognition is believed to be better explained by other contextual factors such as maternal cognitive ability, housing conditions, and nutritional status.

**Added value of this study**

Despite the higher burden of both poverty and risk for unmet developmental potential in LMICs, most research on cognitive development has focused on Western, Educated, Industrialized, Rich and Democratic (WEIRD) populations. Although there is variability across specific settings, conditions in LMICs are often characterized by lower health status achievement in children (e.g., undernutrition, greater incidence of infectious disease, higher prevalence of anemia), decreased access to services (e.g., medical or educational), and more limited infrastructure (e.g., safety of housing conditions, water supply, etc.) which may have differential effects on the ways that poverty impacts developmental outcomes. The current study adds to this body of research to inform prevention and intervention approaches that are targeted, evidence-based, and culturally informed by groups that need them most.

**Implications of all the available evidence**

Our findings suggest that there are increasing disparities from expectations in cognitive development through the first four years of life among children in Bangladesh. Importantly, as the degree of social adversity becomes more severe (i.e., poverty level), the degree and duration of impact on cognitive scores also increases. Our findings provide preliminary evidence for the prioritization of translational intervention efforts. Universal interventions to improve cognitive development trajectories in LMICs may benefit from prioritizing the promotion of nutrient-sensitive interventions and food security. Selective interventions for those living below the poverty line may benefit from promoting maternal education through policies that foster gender-equitable education systems and high-quality parenting interventions. Targeted interventions for cognitive development may prioritize specific housing/environmental risk exposure groups (e.g., children at high risk for cognitive delays due to elevated levels of air pollutants or other toxins in their community).

## Introduction

The number of children globally exposed to poverty and growth faltering is estimated at 43%.^1^ Approximately 250 million children living in low and middle-income countries (LMICs) are at risk for not meeting their developmental potential.^1^ Early childhood is a sensitive period during which aspects of the child's environment, including poverty, can drive biological changes that become embedded in a child's long-term cognitive development.^2^ Much of the existing research in this area has relied on poverty and growth faltering as a proxy for developmental potential, but newer research using parent-report measures of cognitive development suggest that 37% of children between 3 and 4 years of age perform poorly on cognitive and socioemotional domains.^1^

Existing research on early child cognitive development emphasized the need for quality programming that can integrate social determinants including nutrition, security and safety, responsive caregiving, and opportunities for early learning.^3^ Maternal education, and to a lesser extent paternal education, are social determinants that are strongly associated with child cognitive development. Research in the United States suggests that caregiving quality (e.g., maternal stimulation and responsive caregiving) is likely the primary mechanism explaining the link between maternal education and child development.^4^, ^5^, ^6^, ^7^ Paternal education has been studied less than maternal education, and even less work has been done examining its role in LMICs. Emerging research in Ecuador, Colombia, and Bangladesh has found protective effects of combined parental education on child cognitive development.^8^, ^9^, ^10^ One study has examined the role of paternal education in middle childhood, providing evidence that paternal education is only related to visual integration abilities.^10^ Additional research is needed to examine the unique contributions of maternal and paternal education levels on child cognitive development, especially in early childhood, longitudinally, and in diverse cultural contexts.

Housing conditions have also been related to child development outcomes. Poor water, sanitation and hygiene (WASH) conditions are linked to inflammation, stunting, anemia, and small intestinal disorders that can negatively affect cognitive development.^11^ Poor WASH conditions in LMICs have been associated with reduced school attendance due to behavioral changes (e.g., water carrying) or health problems that lead to school absence. The use of biomass fuel, and the associated air pollutant toxicants produced by such fuel, for cooking has also been related to decreased cognitive scores in school-aged children in Nepal.^12^ Liquified petroleum gas (LPG) use has been found to have a mild protective effect on cognitive scores in school-aged children in Ghana, at least partially due to reductions in time away from school to collect biomass fuel.^13^ Household air pollution from biomass fuel use for cooking has been associated with oxidative stress, inflammation, and dopaminergic and glutaminergic impacts on synaptic plasticity.^14^

Other aspects of poor housing conditions (e.g., overcrowding, mold) in HICs increased the risk of physical health problems by 25% during childhood and were associated with lower cognitive scores and school attendance.^15^ There are gaps in the understanding of how these factors may co-occur to produce risk, their salience relative to other factors (e.g., parental education), and whether these effects are noted in early development. Furthermore, household assets (e.g., furniture, appliances, entertainment units) have been used in research as proxies for socioeconomic status, including in low-income communities within LMICs where there may not be sufficient variability in monetary income.^16^ However, whether tangible household assets specifically confer a protective effect for cognitive development outcomes, beyond the effects of other aspects of socioeconomic status like parental education in not well described.

The effects of food security on child development outcomes have been examined in two systematic reviews of studies in HICs. Household food insecurity, even at modest levels of undernutrition, was associated with worse general cognitive and executive functioning through adolescence.^17^^,^^18^ However, a minority of studies (<10%) examine early childhood. Studies have documented the detrimental effects of food insecurity on cognitive outcomes in school-aged children (5–15 years) in South Africa, Malawi, and Ghana, underscoring the importance of developing interventions that deliver additional levels of social protection, for example protections for food security, in addition to cash transfers, to improve outcomes in childhood.^19^ Research is needed in LMICs to determine whether these findings might extend to even earlier periods in development (0–5 years of age) in other settings. Early childhood is a crucial period when the brain is expecting nutrient-sensitive inputs (e.g., protein, long-chain polyunsaturated fatty acids, trace minerals, and vitamins), which if unmet can negatively affect neurodevelopment.^20^

We sought to fill some of the existing gaps in the literature by identifying social determinants that are most related to cognition longitudinally (from infancy through 7 years) and determine whether social determinants may interact with poverty level to increase risk for cognitive delays.

## Methods

### Study design and participants

Participants in the current study were enrolled in the Bangladesh Early Adversity Neuroimaging (BEAN) longitudinal study, designed to measure the effects of exposure to early adversity and child development in low-income settings.^21^ Data were collected from 2015 through 2022. Participants were recruited from medical (hospitals, primary care centers), research (icddr,b), and community-based settings (schools, daycares) in Dhaka City including Mirpur, Dhanmondi, Baridhara, Banani, and Uttara.

To be included in the study, participants had to have been born at ≥34 weeks of gestation, with no known history of neurological abnormalities, traumatic brain injury, genetic disorders, or visual/auditory delays or impairments. Study and home visits took place when the child was 6 months, 2 years, 3 years, 4 years, 5 years, and 7 years. Participants completed approximately three developmental assessments on average, with a total of 1397 cognitive assessments across all participants (*N* = 542) and time points.

Study procedures were approved by the Institutional Review Boards at the icddr,b and Boston Children's Hospital. The child's parent or legal guardian provided written informed consent prior to the initiation of study activities.

### Procedure

Data for the current study were collected through a combination of interviews, home observations, or direct assessments. Due to low levels of literacy and education for some participants in the sample, interviews were conducted verbally in Bangla by research staff that were local and native to the community. Questions used in interviews were field tested with a subsample of families before data collection began to ensure their appropriateness. Language and cultural adaptations were made to the child development assessments. Developmental assessments (Mullen Scales of Early Learning at 6 months, 2 years, and 3 years; Wechsler Preschool and Primary Scale of Intelligence, Third Edition at 4, 5, and 7 years) were administered by psychologists or trained research assistants. Some variables were operationalized using a single item (e.g., maternal education, paternal education, food insecurity) while indices/composites were generated for others (e.g., housing risks, housing assets). For example, food security was assessed by having families rate their household food availability in the past year as in a deficit all year, sometimes in a deficit, neither in a surplus nor a deficit, or in a surplus (with higher scores indicating higher levels of food security). Detailed information about study measures, adaptations, and scoring can be found in the Supplementary Materials (Supplementary text 1).

### Statistical analyses

Statistical analyses were conducted using IBM SPSS Statistics for Macintosh Version 29 (IBM Corp, Armonk, NY, USA) and Python Version 3.12.3. A *p*-value of less than 0.05 (two-tailed level) was considered statistically significant. To characterize cognitive trajectories, growth models were used to capture interindividual differences in intraindividual change over time. Growth models were estimated for the full sample, and separately for those above and below the poverty line. Marginal means for cognitive scores were estimated for each group and timepoint, and pairwise comparisons were Bonferroni corrected to test mean differences.

To determine associations between social determinants of interest and cognitive development, Linear Mixed Models (LMMs) with maximum likelihood (ML) estimation were used. LMMs account for observations that tend to be correlated (e.g., due to repeated measures) and for imbalances across waves of data collection. The LMMs for the current analyses included fixed effects for each of the social determinants of interest and random effects for individual, time, and cohort. The same procedure was used to test for interaction effects.

### Role of funding source

The funders of the study had no role in study design, data collection, data analysis, data interpretation, or writing of this report.

## Results

### Preliminary analyses

Descriptive statistics are provided in Table 1. Participants lived in households where the mean income per capita per day was $3.04 USD (*SD* = 4.41). Approximately 52% of the sample (*n* = 282) were living below the poverty line. Mothers had a mean of 8.36 years of education completed (*SD* = 5.20) and fathers had a mean of 7.74 years of education completed (*SD* = 5.08). Individuals living below the poverty line had a mean income of 92 cents (per capita per day) while those above the poverty line had an income of 5.35 (Mean difference = 4.43, *p* < 0.001). Supplementary Tables S1–S3 describe item-level frequency statistics for the housing risk, household assets, and food security variables. Levels of maternal and paternal education were highly correlated (r = 0.791, *p* < 0.001). At the bivariate level, all social determinants included in models were significantly associated with child cognitive outcomes.

**Table 1:** Descriptive statistics for social determinant variables (n = 542).

	Mean (SD)	Range
Income per capita per day (USD)	3.04 (4.41)	0.27–54.84
Expenses per capita per day (USD)	2.06 (2.18)	0.24–18.37
Income/expenses ratio	1.35 (0.51)	1.00–6.67
Maternal education (in years)	8.36 (5.20)	0–17
Paternal education (in years)	7.74 (5.08)	0–17
Number of housing risks	2.63 (1.23)	0–7
Number of household assets	7.46 (2.24)	1–12
Food security	3.53 (0.76)	1–4

### Trajectories of cognitive development for full sample

**Fig. 1:**
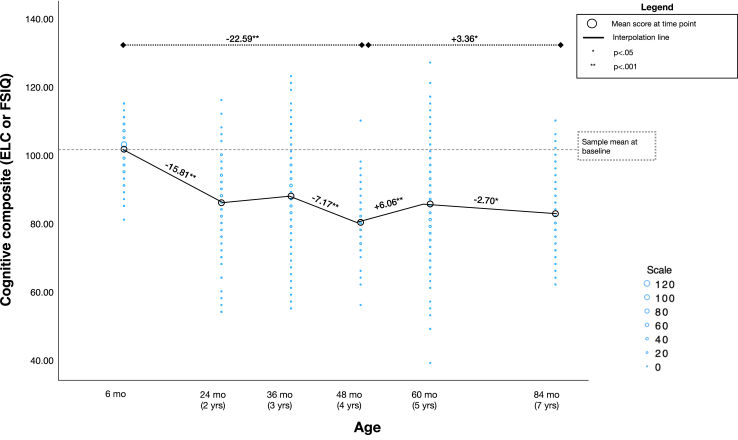
*Cognitive composite standard scores (Early Learning Composite or Full-Scale IQ Score) from 6 months to 7 years of age for the full sample*. Note. Figure displays data for 542 participants with repeated measures for a total of 1397 cognitive assessments from 6 months to 7 years of age. Given the large number of observations, data points are binned and scaled. The scale corresponds to the number of participants.

There was a significant fixed effect of time on cognitive development scores. Estimated marginal means at each time point were 102.63 at 6 months, 86.82 at 2 years, 87.21 at 3 years, 80.03 at 4 years, 86.09 at 5 years, and 83.39 at 7 years. Bonferroni corrected pairwise comparisons indicated a significant drop in scores from 6 months to 2 years (−15.81, *p* < 0.001), no significant changes from 2 to 3 years, and another drop in scores from 3 to 4 years (−7.17, *p* < 0.001). From 6 months to 4 years there was a mean difference of −22.59 (*p* < 0.001). From the 4 to 5 years there was a significant increase in scores (+6.06, *p* < 0.001) and from 5 to 7 years there was another drop in scores (−2.70, *p* = 0.013). From 4 to 7 years there was an overall increase in scores by 3.36 (*p* = 0.02) (Fig. 1).

### Trajectories of cognitive development by poverty level

Among the group of participants living below the international poverty line, there was a significant fixed effect of time on cognitive development scores. Estimated marginal means at each time point were 103.93 at 6 months, 85.76 at 2 years, 88.22 at 3 years, 79.35 at 4 years, 83.85 at 5 years, and 81.86 at 7 years. Bonferroni corrected pairwise comparisons indicated a significant drop in scores from 6 months to 2 years (−18.17, *p* < 0.001), no significant changes from 2 to 3 years, and another drop in scores from 3 to 4 years (−8.88, *p* < 0.001). From 6 months to 4 years there was an overall decrease in scores by 24.58 (*p* < 0.001). From the 4 to 5 years there was a significant increase in scores (+4.51, *p* < 0.001) and from 5 to 7 years there was no significant change in scores. From 4 to 7 years there was no significant difference in scores.

**Fig. 2:**
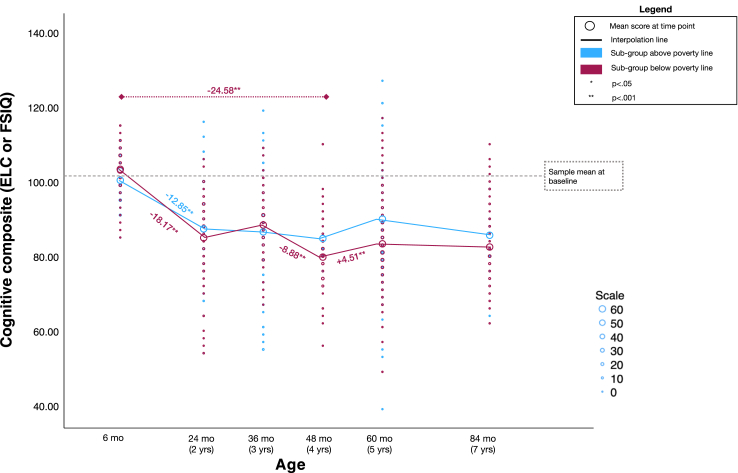
*Cognitive composite standard scores (Early Learning Composite or Full-Scale IQ Score) from 6 months to 7 years of age by poverty level*. Note. Figure displays data for 542 participants with repeated measures for a total of 1397 cognitive assessments from 6 months to 7 years of age. Given the large number of observations, data points are binned and scaled. The scale corresponds to the number of participants.

Among the group of participants living above the international poverty line, there was a significant fixed effect of time on cognitive development scores. Estimated marginal means at each time point were 100.46 at 6 months, 87.61 at 2 years, 86.97 at 3 years, 83.60 at 4 years, 90.33 at 5 years, and 87.28 at 7 years. Bonferroni corrected pairwise comparisons indicated a significant drop in scores from 6 months to 2 years (−12.85, *p* < 0.001) and no significant changes at any of the subsequent time points from 2 to 7 years (Fig. 2).

### Social determinants and child cognition

To determine the associations between social determinant variables of interest and cognitive scores, LMM with random effects at the individual level, time, and cohort level were modeled. In these models, both maternal education (*t* = 2.22, *p* = 0.03) and food security (*t* = 4.48, *p* < 0.001) had main effects on cognitive scores. Higher levels of both maternal education and food security were associated with higher scores on cognitive assessments. Paternal education, housing risks, and household assets were not significantly related to cognitive scores after adjusting for the effects of maternal education and food security (Table 2).

**Table 2:** Longitudinal models for cognitive development from 6 months to 7 years.

Main effects model						
Parameter	Estimate	SE	t	Sig.	95% CI	
Intercept	79.95	2.49	32.10	<0.001	75.05	84.85
**Maternal education**	0.30	0.14	**2.22**	**0.03**	0.03	0.57
Paternal education	0.00	0.14	−0.02	0.99	−0.27	0.27
Housing risks	−0.20	0.36	−0.56	0.57	−0.90	0.50
Household assets	−0.19	0.27	−0.68	0.50	−0.72	0.35
**Food security**	2.75	0.61	**4.48**	**<0.001**	1.54	3.96

Interaction effects with poverty						
Parameter	Estimate	SE	t	Sig.	95% Confidence Interval	
Intercept	78.21	2.57	30.38	<0.001	73.14	83.28
Maternal education (above PL)	−0.08	0.27	−0.31	0.76	−0.62	0.46
**Maternal education (poverty)**	0.62	0.17	**3.76**	**<0.001**	0.30	0.95
Paternal education (above PL)	0.10	0.31	0.34	0.74	−0.50	0.71
Paternal education (poverty)	0.11	0.16	0.71	0.48	−0.20	0.42
Housing risks (above PL)	1.40	0.92	1.53	0.13	−0.40	3.20
Housing risks (poverty)	−0.31	0.38	−0.83	0.41	−1.06	0.43
Household assets (above PL)	−0.30	0.44	−0.68	0.50	−1.17	0.57
Household assets (poverty)	0.06	0.34	0.18	0.86	−0.61	0.72
**Food security (above PL)**	3.25	1.12	**2.90**	**<0.001**	1.05	5.45
**Food security (poverty)**	2.43	0.65	**3.72**	**<0.001**	1.15	3.72

*Note.* Poverty level is defined by the international poverty line standards set by the World Bank. Bolded text indicates significance at <0.05 (two-tailed).

Above PL = >$1.90 USD per capita per day in household.

Poverty = ≤$1.90 USD per capita per day in household.

**Fig. 3:**
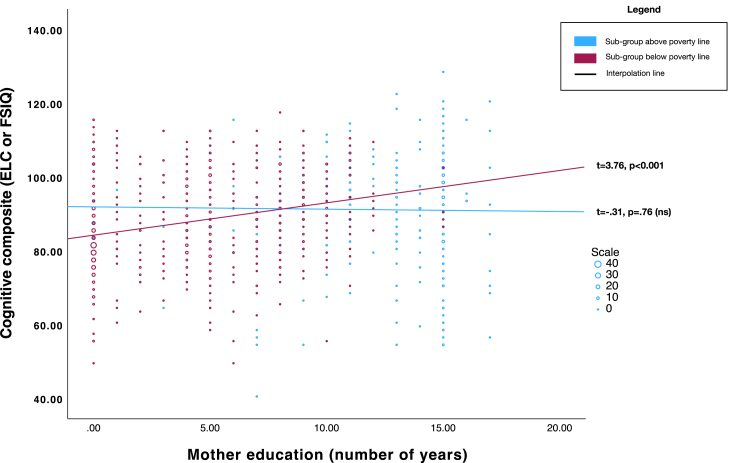
*Interaction effect between maternal education and poverty level on cognitive scores from infancy to 7 years*. Note. Figure displays data for 542 participants with repeated measures for a total of 1397 cognitive assessments from 6 months to 7 years of age. Given the large number of observations, data points are binned and scaled.

A second LMM tested for interaction effects between social determinants of interest and poverty level (Table 2). Maternal education significantly interacted with poverty level, whereby higher levels of maternal education were associated with higher cognitive scores for children living below the poverty line (*t* = 3.76, *p* < 0.001), but not for those living above the poverty line. Food security also had an interaction effect with poverty levels (Fig. 3). Both groups had significant positive associations between food security and cognitive scores, but the effect size for the association between food security and cognitive scores was stronger for those living below the poverty line (*t* = 3.72, *p* < 0.001) than for those above the poverty line (*t* = 2.90, *p* < 0.001). No interaction effects were observed between poverty and paternal education, housing risks, and household assets respectively on the outcome of child cognitive scores after adjusting for the effects of maternal education and food security.

## Discussion

In the current study, we found cognitive scores that increased in deviations from expectations throughout first four years of life, with more extreme differences in children living below the poverty line. In contrast, those living above the poverty line had a relatively smaller divergence during the first two years of life and stable trajectories thereafter. Maternal education and food security had promotive/protective main effects on longitudinal cognitive development for the full sample. Maternal education and food security had stronger effects on cognitive development for children living below the poverty line compared to those above the poverty line.

The increasingly disparate cognitive trajectories observed in this sample in Dhaka stand in contrast to trajectories in WEIRD contexts. In WEIRD contexts, a gap is typically observed in the mean cognition scores between infants with perinatal biological insults (e.g., preterm birth, white matter abnormalities, brain injury) and healthy infants, but both groups’ trajectories remain stable on average through childhood and adolescence.^22^ It is worth noting that the increasing divergence from expectations in cognitive scores observed in Dhaka are consistent with the trajectories of Native American children in the U.S. during the first three years of life, albeit more extreme. Mitchell, Croy^23^ found that at 6 months, Native American infants living in the United States were near the national norms (100) but by 3 years their cognitive scores had dropped almost a full standard deviation. The widening disparities in the current sample were observable within the same assessment method (e.g., MSEL from 6 months to 2 years) and across methods (e.g., MSEL at 3 years and WPPSI at 4 years).

Cognitive skills assessed and which develop throughout infancy tend to rely more heavily on sensorimotor processing.^24^ Consequently, if an infant has not had biological or perinatal insults that impact the functioning of primary sensory areas and motor cortices, they are often able to meet cognitive demands at younger ages (e.g., 6 months) as observed in our sample and Mitchell, Croy^23^ However, past infancy, cognition becomes increasingly reliant on more complex and integrative processing (e.g., identifications of patterns and entities like words or faces).^24^ As children are continuously exposed to poverty or other deviations from an optimal environment and cognitive processes become more integrated, the impacts of poverty on cognition may become more evident. From the current study's findings, it also appears that as the degree of social adversity becomes more severe, the degree of impact on cognitive scores and the chronicity of impact also increases. Some of the full sample trends, like the six-point increase from 4 to 5 years and the three-point decrease from 5 to 7 years also seems to coincide with broader societal transitions that may impact child cognitive development (e.g., school initiation and the Sars-Cov-2 pandemic).

The bulk of existing evidence suggests that differences in cognitive development observed by poverty level are likely attributable to risks that tend to co-exist with poverty, rather than poverty itself.^2^ Importantly, the risk factors that negatively impact cognitive development are copious, complex and interacting (e.g., psychosocial, pathogen exposure, poor food access/quality, environmental contaminants/risks).^2^ Given the diversity and complexity of factors that likely interact within an individual to confer risk for worse developmental outcomes, we sought to isolate which aspects of environmental exposure to poverty are most saliently associated with cognitive development trajectories over the first seven years of life, which in this sample were on average characterized by increasingly disparate differences from expectations starting at infancy and through 4 years of age.

The current findings suggest that higher levels of maternal education are strongly associated with global cognition scores in children between 6 months and 7 years. Importantly, maternal education demonstrated an interaction effect with poverty levels such that higher levels of maternal education were associated with higher cognitive scores for children living below the poverty line, but not for those living above the poverty line. Research demonstrates that even among families with low socioeconomic status, educated mothers are more often engaging in behaviors that are protective for child health and development.^2^ Some examples of protective behaviors include greater adherence to health guidance and engagement in health seeking-behaviors (e.g., taking the child to healthcare visits, seeking medical support when child is sick), enrollment in social welfare programs, and engagement in stimulating activities that promote development.^2^ Protective behaviors are often co-occurring and act on multiple biological systems (e.g., infection, malnutrition, biological stress responses) that impact brain structure and neurocognitive functioning.^2^

Higher levels of food security were also associated with higher cognitive development scores longitudinally. Significant interaction effects with poverty level also suggest that food security may confer a greater protective effect in the context of poverty (i.e., for families living below the international poverty line). Nonetheless, there were significant protective effects of food security for all children in this sample. These findings are in line with extensive work in HICs in school-aged children and adolescents which finds that even modest levels of undernutrition negatively impact cognition and executive functioning.^17^^,^^18^ Our findings emphasize the need for interventions that provide social protections related to food security. Preliminary research suggests this can be accomplished either directly through the provision of food, or a combination of both food provisions and cash transfers; however, additional systematic review work is needed to determine the precise approach to most effectively target food insecurity and the precise mechanisms through which food insecurity/malnutrition negatively impact cognitive development.^19^ Food insecurity is linked to malnutrition (i.e., caloric and micronutrient deficiencies) which may directly impact neural development, and also affect other physiological responses, that in turn adversely impact developmental outcomes.^2^

Paternal education, number of housing risks, and household assets were not significantly associated with cognitive scores after adjusting for the effects of maternal education and food security. Past research in LMICs has found a protective effect of combined parental education and a unique contribution of paternal education to visual integration scores in school-aged children.^8^, ^9^, ^10^ Our findings suggest that in this context where mothers tend to be the primary caregivers, maternal education has a bigger influence on overall child cognition. More work is needed in samples with lower rates of assortative mating, and in contexts where the father is the primary caregiver as paternal education could be a bigger driver of child cognitive ability in those contexts.^25^ Research is also needed to examine the contribution of maternal and paternal education respectively for sub-domains of cognition.^10^

Housing risks and assets were not significant predictors of longitudinal cognitive development after adjusting for other social determinants. Past research has found associations between specific housing risks and lower child cognitive scores among school-aged children.^11^, ^12^, ^13^^,^^15^^,^^26^, ^27^, ^28^ However, this research has documented unadjusted associations that reflect confounding according to systematic review risk of bias gradings.^29^ For example, improved sanitation infrastructure promotes school attendance by reducing water carrying and physical health problems.^26^^,^^29^ Factors like household air pollution may have small statistical effect sizes and clinically insignificant effects at the population level, such that only prolonged and higher doses of exposure negatively influence child cognition.^30^^,^^31^ Taken together, these findings suggest that while housing risks may have less salient effects on child cognitive development at the population level, some environmental risks may still be relevant targets for high-exposure groups or for other outcomes like physical health.^31^

Our findings should be considered with some limitations in mind. Efforts were made during recruitment to include families in the study that were from a variety of socioeconomic levels and to capture a large sample. However, the sample is derived through non-random sampling, which may limit the generalizability of findings. Both a strength and a limitation of our approach is the use of broad social determinants. While the generation of indices (e.g., household assets and risks) allowed us to capture overall risk by categories and identify areas for intervention at the group level, they may occlude risks that have small effect sizes (e.g., air pollution exposure). In contrast, other variables (e.g., food insecurity) relied on a single item which may be prone to recall bias. Future research is also needed to delineate the specific pathways and mechanisms that may lead to worse cognitive outcomes (e.g., malnutrition or levels of child stimulation).

The use of standardized, validated (in Western contexts), and age-appropriate developmental assessments is a strength given that most existing research relies on parent-report or is cross-sectional. Steps were also taken to adapt measures to the study population, reduce reliance on Western norms, and focus the interpretation of findings on relative changes over time, in line with current best practice guidelines. However, large-scale norming and formal linguistic/cultural adaptations of cognitive assessments by test developers is necessary to replicate existing work in LMICs.

### Conclusions

Our findings and those of Mitchell, Croy^23^ suggest that there are increasing disparities from expectations in cognitive development through early childhood among children exposed to higher levels of social adversities. Our findings also suggest that as the degree of social adversity heightens, the severity and chronicity of cognitive delays also increases. The current findings indicate that universal interventions to improve cognitive development trajectories in LMICs may benefit from prioritizing the promotion of nutrition-sensitive interventions and food security. Existing systematic review work suggests that interventions which bolster consumption of or include supplements for multiple micronutrients, iron, and long-chain polyunsaturated fatty acids have the greatest effect on cognitive development in early childhood.^32^^,^^33^ Selective interventions for those living below the poverty line may benefit from promoting maternal education. This can either be accomplished through the promotion of gender-equitable education systems at a policy level and the implementation of high-quality parenting interventions, which current systematic review work suggests are particularly effective (3×) in LMICs compared to HICs.^34^ Future work should also explore whether more targeted interventions for cognitive development should prioritize specific housing/environmental risk exposure groups (e.g., children at high risk for cognitive delays due to elevated levels of air pollutants or other toxins in their community).

## Contributors

CAN and RH contributed to the funding acquisition and study design of the prospective cohort study used for this report. VV conceptualized the current project with input from CAN, EFS, and LMT. FT, SHK, and TS contributed to the collection of data. VV led the data analysis, with support from EFS. All authors except LMT have access to the data. VV wrote the first draft of the manuscript with input from CAN. All authors contributed to the revision of the manuscript. All authors accept responsibility for the decision to submit for publication.

## Data sharing statement

Data are available from the corresponding author upon reasonable request and with permission of the Bill and Melinda Gates Foundation.

## Declaration of interests

We declare no competing interests.
