# Supplementary material for: Trajectories and social determinants of child cognitive development: a prospective cohort study from infancy through middle childhood in Dhaka, Bangladesh
Source: Lancet Reg Health Southeast Asia. 2024 Nov 23;32:100511. doi: 10.1016/j.lansea.2024.100511 (PMC11626075; doi:10.1016/j.lansea.2024.100511)
Supplement: Supplementary Materials [file mmc1.docx]

**Supplementary Materials**

**Supplementary Text 1**

*Detailed Study Procedure and Measure Information*

**Poverty and Parental Education**

Participating families were asked during an interview to report the monthly income (in Bangladeshi Taka, BDT) for each income-bearing member of their household, their household’s monthly expenses, and the number of people living in their household. This information was used to generate a standard income value for each participant (income per capita per day) and converted to United States Dollars (USD) to allow for comparisons with the World Health Organization’s international poverty line. Expenses were also calculated per capita and per day and in USD for each participant following the same procedure. An income-to-needs ratio (income divided by expenses) was then generated. Parental education was assessed with separate questions about the mother and father’s highest level of education achieved (in number of years).

**Housing Risks**

Items included in the family housing risk index were determined through observations during home visits by research staff. The following categories were rated by research staff: flooring (1=earth, 2=bamboo/wood, 3=cement/tiles), walls (1=bamboo/cane/straw/jute stick, 2=rudimentary walls like mud or mixed with mud, 3 =tin wall, 4 = bricks/cement blocks/concrete walls), roofing (1=natural material like straw, 2= rudimentary roofing like polythene or mixed with mud, 3=finished roof like concrete, 4=tin roof), cooking gas (0=access in household, 1=no access in household), toilet type (1=septic tank or toilet, 2=single ventilated improved pit with water seal, 3=water-sealed or slab latrine, 4=flash toilet, 5=poor flash, 6=pit latrine, 7=open latrine, 8=hanging latrine, 9=bush or field as a latrine, 10=ventilated improved pit), toilet sharing (0= Private household toilet, 1= Toilet shared with other households), open drains (0=no open drains beside home, 1=open drain beside home), drinking water source (1=Municipality supply/piped water, 2 =own arrangement by pump, 3=tube well, 4=well/pond/canal), household crowding (number of people in household and number of rooms per household noted).

All housing risks were recoded as binary risks (0 or 1) as shown in Supplementary Table 1. A total housing risk index was then calculated for each participant. Composite scores for housing risks were generated across participants, with higher scores indicating a greater number of housing risks. Possible scores on this index ranged from 0 (no housing risks) to 9 (seven housing risks).

**Household Assets**

Items included in the household assets index were determined through observations during home visits by research staff. Research staff noted the presence of the following items (0=no, 1=yes): telephone/mobile, wardrobe, table, chair, bench, watch or clock, cot or bed, working radio, working television, bicycle, motorcycle, sewing machine, fan. Composite scores for household assets were generated, with higher scores indicating a great number of household assets. Possible scores ranged from 0 (no household assets) to 13 (twelve household assets).

**Food Security**

Food security was assessed during interviews with families. Families were asked to classify their household food availability in the past year according to the following options: 1) food deficit all year, 2) sometimes in a deficit, 3) neither a surplus nor a deficit, 4) surplus. Higher scores on this item indicate higher levels of food security.

***Child Cognitive Development***

Age-appropriate developmental assessments were used to assess child cognitive development from 6 months to 7 years. The Mullen Scales of Early Learning (MSEL) were used to assess cognitive development at 6 months, 2 years, and 3 years. The Wechsler Preschool and Primary Scale of Intelligence, Third Edition (WPPSI-III) was used to assess cognitive development at 4 years, 5 years, and 7 years.

The MSEL is a play-based developmental assessment that measures fine motor, visual reception, receptive language, expressive language and gross motor ability in children from birth to 68 months, although ceiling effects are noted in children that are between 51 and 68 months. The MSEL produces a standard composite score (*M=*100, *SD=*15) also referred to as the Early Learning Composite (ELC) that includes all subscales except gross motor. The WPPSI-III is a measure of cognitive development for children ages 30 to 84 months. For children that are 4 years and older, the WPPSI produces a full-scale score (FSIQ) that encompasses verbal comprehension, visual spatial, fluid reasoning, working memory, and processing speed. Like the MSEL, the WPPSI-FSIQ also has a mean of 100 and a SD of 15.

Both the MSEL and the WPPSI-III were administered by local research assistants and psychologists. The items were translated and culturally adapted through testing that occurred during pre-piloting. Prior studies (e.g., Jensen and colleagues, 2019) have provided a full description of the language, cultural, and scoring adaptations made to the developmental assessments in this study. Subscale scores for cognitive measures (ELC and FSIQ) were calculated by standardizing raw scores using the sample mean and adjusting for age, given that existing norms for the measures are derived from Western populations. Standardized composite scores were then derived, as has been done in prior research with this sample. Prior validation studies for both the MSEL and the WPPSI-III in LMICs suggest strong psychometric properties, including past research in Bangladesh (e.g., Kippler and colleagues, 2012).

Jensen SK, Tofail F, Haque R, Petri Jr WA, Nelson III CA. Child development in the context of biological and psychosocial hazards among poor families in Bangladesh. *PloS one* 2019; **14**(5): e0215304.

Kippler M, Tofail F, Hamadani JD, et al. Early-life cadmium exposure and child development in 5-year-old girls and boys: a cohort study in rural Bangladesh. *Environ Health Perspect* 2012; **120**(10): 1462-8.

**Supplementary Table 1**

*Frequency Statistics for Housing Risks Composite Items (n=542)*

| **Housing Risks** | | N | % |
| --- | --- | --- | --- |
| Flooring | Cement | 535 | 98.71 |
|  | Earth, wood bamboo | 7 | 1.29 |
| Walls | Bricks, cement blocks, concrete | 488 | 90.04 |
|  | Bamboo, cane, straw, mud, tin | 54 | 9.96 |
| Roofing | Finished roof | 336 | 61.99 |
|  | Straw, polyethylene, mud | 206 | 38.01 |
| Cooking gas | Access in household | 453 | 83.58 |
|  | No access | 89 | 16.42 |
| Toilet type | Septic tank or toilet | 340 | 62.73 |
|  | Latrine or bush/open field | 202 | 37.27 |
| Toilet sharing | Private household toilet | 253 | 46.68 |
|  | Toilet shared with other households | 289 | 53.32 |
| Open drains | No open drain | 133 | 24.54 |
|  | Open drain in front of home | 409 | 75.46 |
| Drinking water source | Municipality supply | 531 | 97.97 |
|  | Non-municipality | 11 | 2.03 |
| Household crowding | < 3 people per room in household | 383 | 70.66 |
|  | > 3 people per room per household | 159 | 29.34 |

**Supplementary Table 2**

*Frequency Statistics for Household Assets Composite Items (n=542)*

| **Household assets** |  | N | % |
| --- | --- | --- | --- |
| Telephone/mobile | No | 11 | 2.03 |
|  | Yes | 531 | 97.97 |
| Wardrobe | No | 142 | 26.20 |
|  | Yes | 400 | 73.80 |
| Table | No | 227 | 41.88 |
|  | Yes | 315 | 58.12 |
| Chair | No | 169 | 31.18 |
|  | Yes | 373 | 68.82 |
| Bench | No | 432 | 79.70 |
|  | Yes | 110 | 20.30 |
| Watch or clock | No | 89 | 16.42 |
|  | Yes | 453 | 83.58 |
| Cot or bed | No | 11 | 2.03 |
|  | Yes | 531 | 97.97 |
| Working radio | No | 522 | 96.31 |
|  | Yes | 20 | 3.69 |
| Working television | No | 45 | 8.30 |
|  | Yes | 497 | 91.70 |
| Bicycle | No | 459 | 84.69 |
|  | Yes | 83 | 15.31 |
| Motorcycle | No | 476 | 87.82 |
|  | Yes | 66 | 12.18 |
| Sewing machine | No | 414 | 76.38 |
|  | Yes | 128 | 23.62 |
| Fan | No | 5 | .92 |
|  | Yes | 537 | 99.08 |

**Supplementary Table 3**

| **Food security** | | **N** | **%** |
| --- | --- | --- | --- |
| In the past year | Deficit all year | 12 | 2.21 |
|  | Sometimes in a deficit | 51 | 9.41 |
|  | Neither deficit nor surplus | 117 | 21.59 |
|  | Surplus | 362 | 66.79 |

*Frequency Statistics for Food Security Item (n=542)*

**Supplementary Figure 1**

*Poverty, Maternal Education, amd Paternal Education Level Distributions*
